# Connectivity Fingerprints: From Areal Descriptions to Abstract Spaces

**DOI:** 10.1016/j.tics.2018.08.009

**Published:** 2018-11

**Authors:** Rogier B. Mars, Richard E. Passingham, Saad Jbabdi

**Affiliations:** 1 Wellcome Centre for Integrative Neuroimaging, Centre for Functional MRI of the Brain, Nuffield Department of Clinical Neurosciences, John Radcliffe Hospital, University of Oxford, Oxford, UK; 2 Donders Institute for Brain, Cognition and Behaviour, Radboud University Nijmegen, Nijmegen, The Netherlands; 3 Wellcome Centre for Integrative Neuroimaging, Department of Experimental Psychology, University of Oxford, Oxford, UK; 4 Wellcome Centre for Human Neuroimaging, University College, London, London, UK

**Keywords:** connectivity, brain organization, gradient, individual differences, comparative anatomy

## Abstract

Fifteen years ago, Passingham and colleagues proposed that brain areas can be described in terms of their unique pattern of input and output connections with the rest of the brain, and that these connections are a crucial determinant of their function. We explore how the advent of neuroimaging of connectivity has allowed us to test and extend this proposal. We show that describing the brain in terms of an abstract connectivity space, as opposed to physical locations of areas, provides a natural and powerful framework for thinking about brain function and its variation across the brains of individuals, populations, and species.

## Highlights

The concept of the connectivity fingerprint was described 15 years ago to suggest a crucial relationship between the connections (functional integration) and function (functional segregation) of an area.

The advent of neuroimaging now allows for this concept to be applied on a much larger scale, describing brain organization in terms of a connectivity space.

Describing brain organization in this space is closer to its functional organization and can predict differences in functional activation and behaviour.

By abstracting away into connectivity space one can compare brains across individuals and species.

Comparing differences in the connectivity fingerprints of areas or subareal units in terms of gradients, one can investigate which dimension of connectivity space is relevant for understanding particular aspects of brain organization.

## The Connectivity Fingerprint Defined

Functional brain organization is often described in terms of the principles of segregation and integration [1]. Segregation means a focus on mapping unique **brain areas** (see Glossary) and ascribing functions to each, while integration describes the interactions and relationships between remote neural populations that are required for complex behavior. Although both principles on their own are valuable, a full understanding of the brain requires both. Fifteen years ago, Passingham *et al.* suggested that the concept of a **connectivity fingerprint** could link these two principles [2]. They proposed that the function of each individual area is determined in the main by its unique set of connections with the rest of the brain.

Thus, an area is described within the space of connections. The paper used multidimensional scaling to show that each area occupies a unique place within this space. In the original paper it was suggested, but not proven, that the functions of each area depend on the location of that area in this space. The reasoning was that each area performs a transformation from inputs to outputs and that this transformation depended on the information it received and the areas that it could influence. At the time, connectivity was studied almost exclusively using invasive **tracer** data in non-human species (Box 1). Although the authors suggested that the same conceptual framework could be applied to data acquired through neuroimaging, the paucity of data at the time meant that this extension of the original hypothesis could not be tested.

Box 1 **Historical Overview of Connectivity Research Methods**

The development of the concept of the connectivity fingerprint has been due in no small part to the development of new methods for studying brain organization [81]. Advances in microscopy meant that the brain could be divided up into areas based on differences in cytoarchitecture and myelination, and it later turned out that damage to different parts of the brain led to distinct behavioral impairments. The development of tracers in the early 1960 also meant that the connections between these areas could be studied in detail [82].

In the early 1990 the relationship between these two streams of research, areal specialization and exchange of information, was formalized as the principles of segregation and integration in the brain 1, 83, 84. With the increasing availability of data from tracer studies, it could be appreciated that different areas had unique characteristics [85]. A further step was taken when it was appreciated that there was topography in the areal connections [86]. Large-scale collations of tracer data, such as the CoCoMac database for macaque tracer data 87, 88, meant that these relationships between the connectivity profiles of areas could be analyzed statistically [89]. This prompted Passingham *et al.* [2] to argue that each area had a unique pattern of connections and to argue that neuroimaging might be used to characterize the functional profiles of the different areas.

The 2002 paper provided one of the inspirations for the current movement of mapping the human connectome [90]. Large datasets are easily obtained from neuroimaging and this has inspired the application of many techniques from network science to the description of the brain [91]. In turn, these techniques have made connectivity research suitable for many applications, opening the door for the connectivity fingerprint as a tool in fundamental and clinical neuroscience 43, 92.

Alt-text: Box 1

Since the original paper, the field of connectivity research has taken off in an unprecedented manner 3, 4. Techniques such as **diffusion magnetic resonance imaging (MRI) tractography** and **resting state functional MRI (fMRI) covariance** allow mapping of the connectivity of an individual’s brain in minutes. Connectivity fingerprints can now be defined easily and related to a wide array of variables given the development of tools for fast data acquisition and advanced connectivity analyses 5, 6, 7, and the wide availability of high-quality datasets from both healthy and clinical populations as well as different species 8, 9, 10.

Here, we show that the connectivity fingerprint and its relation to function has become a valuable window into understanding the anatomical and functional organization of the brain. It is now routine to use neuroimaging to describe connectivity fingerprints at the level of the **voxel**, and to do so across areas, networks, individuals, and species.

## Connectivity as a Dimension of Brain Organization

The original paper took the term fingerprint from the unique receptor profiles of areas that were established by Karl Zilles and colleagues [11]. Although neuroimaging has investigated the connectivity of areas as defined by **cytoarchitecture** [12], it has become common to identify regions based on changes in connectivity at the voxel level. **Connectivity-based mapping** of areas has become a major part of cortical cartography (Box 2; [13]).

Box 2 **Cortical Cartography Using Connectivity-Based Parcellations**

Each area has a unique connectivity fingerprint that is critical in determining its function. Passingham *et al.* [2] illustrated their point using meta-analyses of tracer data obtained in the macaque, showing that no regions in prefrontal or premotor cortex showed a similar pattern of connections to the rest of frontal cortex. However, postulating the idea of an areal connectivity fingerprint suggests that the border of a cortical area can be demonstrated by a change in the connectivity profile.

Early approaches [93] tested whether functional connectivity can be used to detect areal borders. They compared the whole-brain connectivity fingerprint of an area in inferior parietal cortex with that of increasingly distant areas. They showed a sharp transition in connectivity patterns, rather than a consistent decline with distance from the seed, and argued that this demonstrates borders between areas. Mapping of boundaries in this way can be used to parcellate the entire cortical sheet [94].

An alternative parcellation approach exploits the unique profile of areas by grouping voxels based on their similarity in connectivity. Employing the at the time newly established technique of diffusion MRI tractography, this approach was originally used to identify two medial frontal areas overlapping with the supplementary and presupplementary motor areas [95]. This approach has since become well established, with high test–retest reliability [96] and consistency across groups 97, 98. Where results could be compared to existing cytoarchitectonic maps, the overlap was high 99, 100, affording the confidence to parcellate regions of the brain for which no prior anatomical investigation was attempted 101, 102, often showing high correspondence with functional regions 103, 104. Most recently and most dramatically, functional connectivity in combination with MRI sequences sensitive to structural markers and task activations has been used to produce a new map of the human cerebral cortex [76].

Alt-text: Box 2

However, the main innovation of the concept of the connectivity fingerprint as used in neuroimaging has been to classify areas not in terms of their physical location but in terms of their connectivity. The point can be illustrated with reference to the extrastriate body area (EBA). This area was originally identified as a cortical region that responded to images of the human body as compared to control stimuli [14]. It was viewed as a purely perceptual area [15]. It was taken on the basis of its location to be one of the areas in the ventral perceptual visual stream that projects into the inferior temporal lobe, rather than one of the areas in the dorsal action stream that projects to the posterior parietal cortex [16]. However, more recent evidence from functional imaging shows that the EBA is active when subjects need to incorporate information on the posture of their own body into an action plan, even in the absence of any visual stimuli related to the body [17]. This prompted the hypothesis that the connectivity of the EBA is reminiscent of a dorsal stream area, rather than a ventral stream area [18]. As expected, compared to the fusiform body area, the EBA shows greater functional connectivity with the dorsal stream. Moreover, a classifier trained to dissociate dorsal and ventral stream areas based on their connectivity assigned the left EBA as being closer to the dorsal stream than areas in its physical proximity.

This result illustrates the point that quantifying an area in terms of its connectivity is critical when trying to understand its function. The point can be generalized beyond specific patterns such as the distinction between dorsal and ventral stream connectivity. One approach consists of finding generic dimensions of connectivity [19]. In such a space, physical distances represent brain connections, in the sense that brain locations that are near each other in connectivity space are more connected to each other, that is they have resting state time series that correlate more closely. Such a space can be defined using results from **spectral graph theory** [20]. The dimensions of connectivity are defined as the **eigenvectors** of the connectivity graph Laplacian 21, 22.

**Figure 1:**
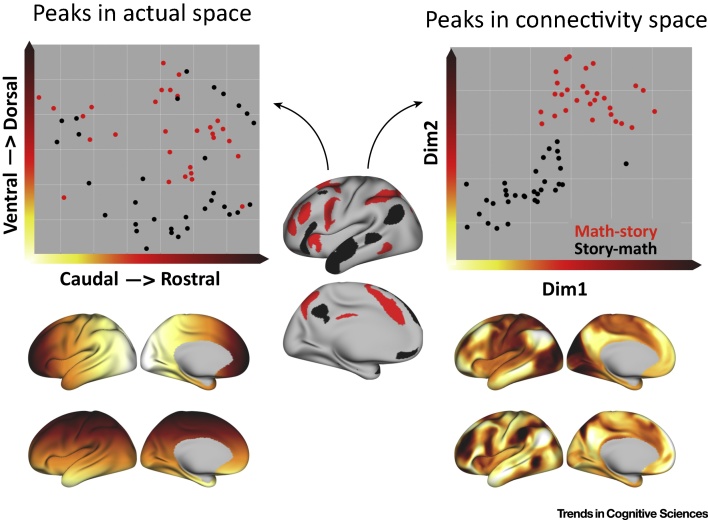
Functional Organization in Physical and Connectivity Space. Group average peak activations for the Human Connectome Project (Q1200 data release). The peaks for two contrasts in a language task (language comprehension vs arithmetic calculation) [78] are scattered in actual, physical space (top left) but highly clustered in connectivity space (top right). In this case, the connectivity space was defined on the basis of resting-state connectivity. Areas that are in close proximity in connectivity space have more similar resting state time courses. The bottom row shows the two dimensions of each space in relation to the cerebral cortex.

We used these methods in Figure 1. This compares brain activity as represented in physical space and brain activity as represented in connectivity space. Comparing local peaks of activity for two different task contrasts, the peaks appear to be scattered around various parts of association cortex with no obvious organization in physical space, but they form clear and distinct clusters in connectivity space. This suggests that the connectivity dimensions form an abstract space that is more closely related to differences in function. The results in Figure 1 demonstrate that it is possible to quickly characterize connectivity fingerprints and to relate function to connectivity space (see also [23]). A particularly potent demonstration is presented by Guntupalli *et al.* [24]. They argued that variance in the organization of connections at the subareal level presents a source of individual variability that is commonly ignored. They therefore expressed each voxel in a particular brain region in a high-dimensional connectivity space. Then, using a matrix rotation, they aligned the different brains into a common connectome space. Importantly, brains registered using this approach allow much improved across-brain classification of response profiles. This demonstrates the power of representing data in this space.

## Does Connectivity Shape Functional Activation?

The previous section showed that describing areas in terms of their connectivity helps organize them according to the criteria that are of most interest in cognitive neuroscience: function. However, as already mentioned the claim made in the original paper was stronger than that. It was that the connectivity fingerprint is a major determinant of the function of an area. In their terminology, connectivity fingerprints relate to functional fingerprints [25].

One way to determine whether connectivity and function are directly related is by exploiting individual differences, testing whether anatomical connectivity predicts variance in function. One study focused on the fusiform face area (FFA). Using diffusion MRI tractography, the authors determined the connectivity fingerprint for each voxel within the FFA with a set of target areas in other parts of the cortex. The authors then established the relationship between functional activation to faces in each voxel and its connectivity fingerprint and showed that this relationship was consistent, such that an individual’s connectivity fingerprint could predict the pattern of functional activation [26]. A subsequent study extended this result to multiple visual categories, demonstrating the ability of the connectivity fingerprint to predict both within- and between-subject variance in functional activation [27].

Impressive as these relationships between connectivity and functional activation are, they are not sufficient to demonstrate a causal relation between connectivity fingerprints and function. In an attempt to establish the relation, one study [28] scanned children before and after they had learned to read. Before they could read, the visual work form area (VWFA) in ventrolateral temporal cortex showed no differential activation in response to faces, letters, or false fonts, even though nearby areas showing selective responses to faces could already be identified. However, after the children had learned to read, the VWFA showed greater activation in response to words, compared to faces, scrambled words, and objects. And the pattern of connectivity of voxels in the occipitotemporal cortex before learning was predictive of the spatial profile of word selectivity after learning. This result establishes that connectivity sets a limit for the processing that can occur within a population of neurons.

The predictive ability of connectivity across individuals has subsequently been explored at the whole brain level. Finn and colleagues used functional connectivity matrices of 128 cortical areas to predict the identity of individuals across scanning session out of a pool of 126 subjects [29]. Comparing the predictive power of different cortical networks, they demonstrated that two networks comprising the medial frontal and frontoparietal association cortex had the most predictive power; presumably due to their higher variation across individuals in general. They also demonstrated that the connectivity profiles of individuals were predictive of their fluid intelligence. This latter result was extended by a demonstration that functional connectivity in a single network is predictive of a range of lifestyle factors [30].

Since the reason that areas are connected is to facilitate information between them, connectivity at rest should predict how much the activation of two areas relates to one another. If connectivity reflects how much information can be passed on to another region, then the activity profile of each region should reflect the activation pattern of all other regions weighted by their connectivity. This is indeed what a recent study found. Both in simulated and actual data, the pattern of the functional activation of a region during tasks could be predicted based on the activity in other regions weighted by the connectivity fingerprint [31]. In a further test of this principle, frequency characteristics of connectivity could be understood in terms of the underlying connectional anatomy [32].

It was recently demonstrated that these results can be generalized [33]. Rather than focusing on predefined areas, this study aimed to predict activation in each **vertex** of the cortical surface across a range of tasks using measures of connectivity. Using an observer-independent approach, the authors used functional connectivity to define homologous **cortical seeds** across individuals that could be used to create connectivity maps. These maps, together with structural markers such as cortical myelin, were incorporated in a model that was trained to predict task activation. Not only was this model successful in predicting the location of activation during tasks, it also captured differences in the amplitude, extent, and shape of the activation in individuals. A model trained on healthy subjects could also predict variations in activity in unseen controls and presurgical neurological patients [34].

**Figure 2:**
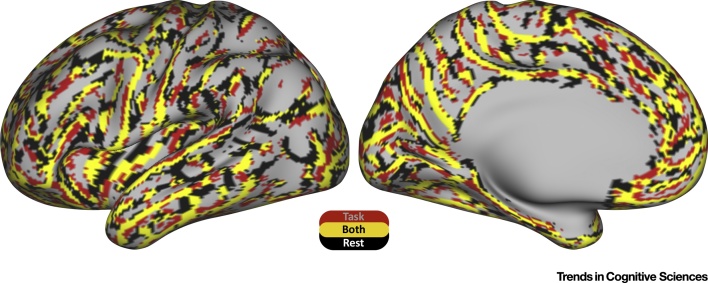
Comparison of Anatomical and Functional Borders. Using data from the Human Connectome Project [10], the borders on the cortical surface were based on changes in connectivity profiles as determined using resting state functional magnetic resonance imaging [79] (black) or by similarities in functional activation during tasks [80] (red). These two borders show strong overlap, demonstrating the strong relationship between the connectivity fingerprint of an area and its functional profile. The borders were calculated using a searchlight approach (unpublished). The cortical hemisphere is parcellated into 200 small contiguous random patches. In each patch spectral clustering is used to identify two clusters and the border between the two clusters is recorded. By repeating the procedure for a number of random brain parcellations we can obtain a map of persistent borders, that is vertices that can be repeatedly identified as belonging to a border between two clusters.

Together, these results support the original proposal that the connectivity fingerprint of an area can be used to predict its function. More importantly, they demonstrate how the concept of connectivity has moved from a descriptor of a single brain region to a measure to predict variability across individuals. From a descriptor, it has become a predictor. However, there is a more direct way of demonstrating that connectivity space and functional space are related. This is to see whether the borders between areas in connectivity space match the borders between areas in functional space. We therefore determined borders based on functional connectivity and, separately, on task-based activation. The results are shown in Figure 2. It can be seen that there is a high similarity of the borders as defined by the two approaches.

## The Connectivity Fingerprint as a Diagnostic in Comparative Anatomy

Connectivity fingerprints can be compared across the brains of different individuals but also across the brains of different species. Comparative neuroscience using traditional histological approaches is laborious, but neuroimaging of different species *in vivo* 35, 36 or post-mortem [37] provides readily available data that allow new comparisons of cortical maps across species. Identifying homologous areas across brains requires measures that are diagnostic of an area [38]. The connectivity fingerprint fits that criterion.

**Figure 3:**
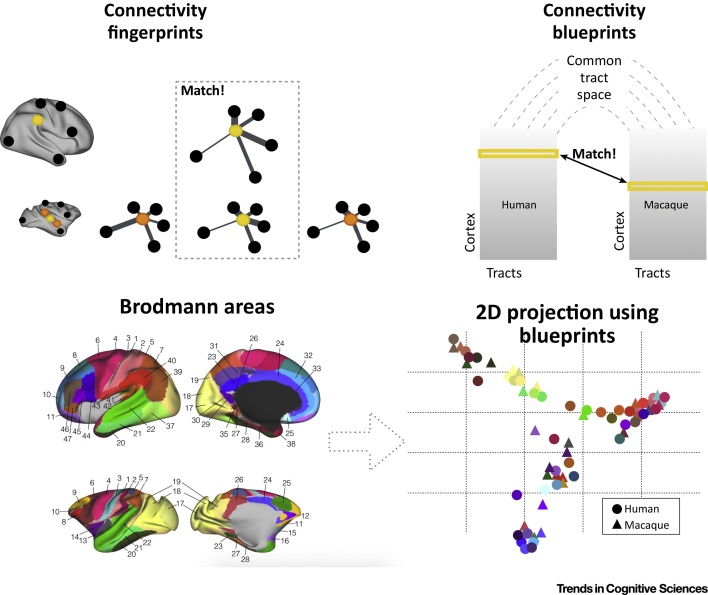
Connectivity Fingerprints as a Diagnostic in Comparative Neuroscience. (top left) Connectivity fingerprint matching. If one has a hypothetical area in the human brain (yellow sphere) that one wants to compare to a number of candidate areas in the macaque (yellow/orange spheres), one can create a connectivity fingerprint with areas that are known to be homologous across the two brains (black spheres). This allows one to abstract away from the particularities of the two brains and identify the best match in connectivity space. (top right) This approach can be generalized by creating connectivity blueprints of the two brains describing each vertex of the cortex in the rows with each of the major white matter tracts in the columns. Since the tracts are homologous across species, they form a common connectivity space, allowing one to match the connectivity fingerprint of any vertex in one brain with that in the other brain (yellow rows). (bottom row) The connectivity blueprint approach can also be used to describe the connectivity fingerprint of cortical areas in two brains and project them on a 2D space, illustrating how close regions of the two brains are in connectivity space. For instance, medial frontal and early visual areas of both species tend to cluster together, while macaque BA7 is closer to inferior parietal human BA40 (data from [50]).

Based on this rationale, one can compare the organization of the human and macaque monkey brain using connectivity fingerprints (Figure 3, top left). One study focused on the dorsal prefrontal cortex and compared areas in the human brain defined by a connectivity-based parcellation with areas defined based on cytoarchitectonic atlases in the macaque monkey [39]. Using resting-state fMRI in both species, connectivity fingerprints were defined for each frontal area with areas across the whole brain whose homology across species had already been established on the basis of cytoarchitectonic criteria. The authors then calculated a measure of the difference between each human and each macaque area, thus identifying which areas were most likely to be equivalent in the two species.

In a modification of this approach, the connectivity fingerprint of the human temporoparietal junction area (TPJ), a region often activated in social cognition paradigms, was compared to the connectivity fingerprint of each voxel in the macaque temporal cortex, in effect searching for a potential match across that entire patch of cortex [40]. This demonstrated that the TPJ has connectional similarities with an area in the middle part of macaque superior temporal sulcus that is sensitive to social information from faces and bodies [41] and that is affected by manipulation of the social environment of macaques [42].

Subsequent work has exploited this approach to systematically compare the organization of the frontal cortex between humans in macaques 43, 44, 45. Most areas in the human frontal cortex could be matched with a macaque area, but there was one notable exception. The human brain contains a lateral frontal polar region that has no preferential match with any macaque area [45]. This region was simultaneously identified in a cytoarchitectonic study that also demonstrated its differential connectivity profile compared to the medial part of the frontal pole [46]. This area shows preferential activation in the type of higher-level decision making tasks [47] that only humans can perform 48, 49.

Matching connectivity fingerprints across species demonstrates the advantages of using neuroimaging to study brain organization. By obtaining whole brain data it becomes possible to build representations of the connectivity fingerprint of any given region with any other region. As was the case for the individual differences approaches described in the previous section, this allows one to abstract away from unimportant idiosyncrasies of any species brain into a dimension that is relevant.

The limitation of using connectivity fingerprints to compare areas across species is that any comparison between areas is dependent on the accuracy of the diagnostic fingerprint. If any of the target areas used to create each fingerprint are not homologous across species, this would throw doubt on the validity of the comparison across species. This does not pose a problem when studying a well-characterized model such as the macaque monkey, but any comparison with other species, including such closely related animals as the great apes, is quickly hampered by our lack of knowledge concerning some of the potential target regions. To circumvent this problem, a recent study compared primate brains using the main white matter fibers as the target of the connectivity fingerprints [50].

The rationale behind this approach is that, although the cortical projections of any major white matter bundle might differ across species 51, 52, the core of these bundles can be identified reliably on diffusion-weighted MRI scans and, hence, suitable tractography protocols can be developed that both identify the tracts but allow an unbiased characterization of their termination points 53, 54. So as to decrease reliance on *a priori* knowledge even more, the authors created a connectivity blueprint describing the connectivity fingerprint of each vertex of the cortical grey matter with a series of 39 tracts. Because the tracts are assumed to be homologous, the blueprints present the connectional organization of the two brains in a common connectivity space. Each vertex could then be matched with each other vertex in the brains of other species (Figure 3, top right). This approach has proved powerful enough to identify areas that are in a different spatial location but nevertheless have a similar connectivity profile across the two brains, such as the motion sensitive area MT+. This lies in the fundus of the superior temporal sulcus in the macaque brain but is located more ventrally in the human brain. In a similar vein, the authors were able to compare all areas of two cortical maps between the two species (Figure 3, bottom row).

This approach can also be used to quantify which parts of the brain have more or less similarity in general to any part of the other brain, thus allowing researchers to search for specializations. When expressing each part of the human cortex in terms of how well it matches any part of the macaque cortex, we found that the posterior temporal cortex has a connectivity fingerprint seen nowhere in the macaque brain [50]; driven in large part by the posterior extension of the arcuate fascicle in the human brain 55, 56. In the macaque brain this connects the parietal cortex and area Tpt with Broca’s area [57], whereas in the human brain it also has its origin in the middle temporal gyrus. This difference is relevant to function since the middle temporal gyrus is activated when people understand what they hear or read [58]. Indeed, development of the arcuate fascicle is crucial for the development of language comprehension [59].

## Beyond Areal Connectivity Fingerprints

The connectivity fingerprint as originally defined describes connectivity at the level of the cortical area. However, it could be argued that this approach ignores key features of anatomical connections, such as their spatial arrangement or **topography** [60]. As we have shown above, some of the most useful applications of the connectivity fingerprint in neuroimaging have applied it at the smallest measurable unit, defining connectivity fingerprints not on the basis of the connections between areas, but on the basis of a single voxel or vertex to many other voxels. In a topographic analysis, the goal is not to look at individual fingerprints, but to look at the relationships between fingerprints as defined at any given level.

One can look for topographic organization of connectivity at both a lower (within an area) and higher (across areas) level of description than the areal fingerprint. Jbabdi *et al.* [60] described some examples of topographic organization within an area, such as in entorhinal cortex where there is convergence between all-to-all mapping from the perirhinal cortex and a **gradient** of increasing connections from the parahippocampal cortex [61]. This arrangement might serve to optimize integration of information in the entorhinal cortex.

Another example is provided by the early visual cortex. Here, different organizational principles, such as **eccentricity** and **polar angle**, overlap. Some of these intricacies might be responsible for a tractography-based parcellation showing unexpected results [62]. Novel techniques aimed at elucidating overlapping gradients of connectivity change are able to recover both the visual eccentricity and polar angle organizations in V1 [63].

That there is structure in connectivity at levels higher than that of the connectivity fingerprint of an individual area was already appreciated by the authors of the original connectivity fingerprint paper 2, 64. They demonstrated that there are connectional families of areas that, although each has a unique fingerprint, show similarities in their connections that mimic their similarity in function. A number of recent studies have exploited the literature of macaque tracer studies to perform hierarchical analyses of the connectivity of cortical areas. They showed that individual regions in the frontal cortex tend to cluster together into groups of areas with similar anatomical and functional properties [65]. Combining these results with similar analyses of parietal cortex, they demonstrated that frontal and parietal groups of areas show a topographic organization in which areas within one group in the frontal cortex tend to connect to areas in a parietal group with a similar function 66, 67. A recent study using neuroimaging in the macaque showed that such results can also be obtained using resting-state functional connectivity (S. Vijayakumar et al., unpublished), opening the way for analyses in a larger range of species.

In both the sub- and supra-areal examples described above, the connectivity fingerprints of smaller units – voxels or areas – are compared to see which aspect of them changes in a meaningful way when moving along a spatial dimension. This type of analysis can be formalized by looking at gradients of connectivity. Such gradients can, for example, be demonstrated for the connections between the striatum and the neocortex. Tracer studies have shown that striatal connectivity with the cortex is organized topographically, although with overlap [68]. This same pattern can be demonstrated using functional connectivity data [69], showing a first level of connectivity organization of the striatum that delineates separate areas such as the caudate, putamen, and nucleus accumbens, but also a second gradient of connectivity change between the ventral striatum and medial frontal cortical areas. Crucially, this connectivity gradient also has functional consequences. Individual differences in the gradient explain individual variations in behavior and do so to a greater extent than parcellation into cortical areas. Thus, by investigating how a connectivity fingerprint changes along parts of the brain, it is possible to isolate specific anatomical features that are relevant to behavior.

The approach of describing cortical connectivity in terms of gradients has been taken furthest in a study describing the connectivity of the whole cortical sheet in terms of two overlapping gradients [19]. The first describes an axis from unimodal to multimodal cortex, and the second dissociates between different modalities, such as visual and auditory cortices. The authors describe these gradients as the core organizing principle of the cerebral cortex, and suggest it provides a grounding of cortical function within the constraints of anatomy [70].

Thus, by looking at systematic changes in the connectivity fingerprint either within a region or across regions one can discern important aspects of anatomical organization. Building on the idea of representing cortical organization in a connectivity space, this approach investigates which dimensions of this space are relevant to understand a particular aspect of brain organization or its relevance to behavior.

## Concluding Remarks

When Passingham *et al.* [2] put forward the concept of the connectivity fingerprint 15 years ago, some of their claims could not be formally tested. However, it has now become possible to do so given that connectivity research has entered the era of big data. In particular, it is now possible to test the link that was assumed between connectivity and function. We can also now examine the principles that underlie interindividual variations in connectivity and function.

The boom in connectivity research, especially in the human brain, is largely driven by the availability of fast data acquisition protocols and the tools to analyze these data. However, the main tools used have not been without their critics. It has been argued that tractography algorithms for diffusion MRI data can produce ambiguous results [71], and it is difficult to determine the grey matter termination points of white matter tracts [72]. However, many of the aspects of connectivity used today, such as the connectivity-based parcellation or studies of patterns of white matter organization, are not necessarily affected by these concerns or can be dealt with using specific tractography strategies [50]. Nevertheless, connectivity fingerprints as established using imaging need to be interpreted with caution and the results cannot always be directly related to results obtained using traditional methods such as tracers 73, 74.

Further development is needed to establish the anatomical validity of the indirect measures provided by neuroimaging [75], but it is critical to emphasize that connectivity as measured using neuroimaging provides meaningful, replicable signals that, crucially, can capture information on the whole brain. This allows the recent development of looking at topographies of connectivity to explore which aspect of connectivity space is relevant to understanding any particular aspect of behavior, as we have described above.

Imaging also allows one to collect data from multiple modalities. Describing imaging data in multiple relevant spaces will increase sensitivity, as demonstrated by a new cortical parcellation based on connectivity, task-based activations, and markers of cortical myelin, describing 180 separate cortical areas [76]. In comparative neuroscience and research looking at individual differences, comparing brain organization across different spaces allows one to test whether principles obtain in one space hold for another. If this is not the case, it might indicate neural reorganization (see Outstanding Questions) [77].

We set out to establish how the concept of the connectivity fingerprint has fared in the 15 years since it was first proposed. Not only is the concept alive and well, the idea of units of brain organization or function, whether voxels, areas, or networks, as embedded within a connectivity space has become ubiquitous in the cognitive neuroscience way of thinking. Thus, the connectivity fingerprint now provides one fundamental way of thinking about the brain.

Outstanding Questions How do we reconcile areal models and within-area organization? Is the areal model a good model or should everything be thought of in terms of a continuum?

How does variation in the topography of connections at the subareal level contribute to individual differences in behavior? Can we find ways in which the integration of different gradients of connectivity within an area contributes to its information processing?

What are the relevant dimensions of a connectivity space and how does one determine them? Can we interpret gradients in connectivity as changes along one or more dimensions of connectivity space?

Can differences in connectivity across individuals be related to complex, multidimensional phenomena such as psychiatric disorders?

How does alignment of different brains into a common connectivity space compare to alignment based on other factors? Can aligning brains based on different spaces be used to compare brain organizations at different levels?

Can we understand differences in connectivity fingerprints across individuals, ontogeny, and phylogeny, within a single framework, given that areas that show great individual variation tend to develop late and are more likely to be unique in the human lineage?

## Glossary

**Brain area**: subpart of the brain defined on an anatomical basis, usually a distinct cytoarchitecture, myelin architecture, and/or receptor architecture, generally associated with a distinct function.
**Connectivity-based mapping**: identification of areas by grouping together of grey matter locations that share similar connectivity profiles.
**Connectivity fingerprint**: unique set of connections to other parts of the brain of any unit of brain organization.
**Connectivity gradient**: changes of connectivity profile as one continuously moves along the grey matter.
**Cortical seed**: area of the cortex taken as the origin of a connectivity analysis.
**Cytoarchitecture**: study of cellular organization of the brain.
**Diffusion MRI tractography**: technique for inferring white matter trajectories based on MRI measurements of anisotropic water diffusion.
**Eccentricity**: in vision, distance from the centre of the visual field.
**Eigenvector**: characteristic vector of a linear operator that only changes by a scaling when that transformation is applied to it.
**Gradient**: increase or decrease in a property, for instance in connectivity to a set of areas.
**Polar angle**: in vision, angle around the centre of the visual field.
**Resting state fMRI covariance**: measure of brain connectivity that relies on idle brain activity, with no explicit task, using fMRI. Connectivity between areas is assessed by looking at the similarity in time courses of the spontaneous brain activity.
**Spectral graph theory**: study of graphs based on the properties of the eigenvectors and eigenvalues of their associated matrices.
**Topography (of connections)**: spatial pattern of connections. Axons that connect a region to its target often follow some sort of spatial arrangement, and connections to different target regions may have different arrangements.
**Tracer**: measurement of neural connectivity dependent on the visualization of axonal transport of substances inserted into the brain.
**Vertex**: smallest unit of resolution of a discrete surface mesh.
**Voxel**: smallest unit of resolution of a MRI data acquisition 3D grid.
